# Combating Cholera

**DOI:** 10.12688/f1000research.18093.1

**Published:** 2019-04-30

**Authors:** Brian Y. Hsueh, Christopher M. Waters

**Affiliations:** 1 Microbiology and Molecular Genetics, Michigan State University, East Lansing, MI, 48824, USA

**Keywords:** cholera, Vibrio cholera, antibiotics, oral-rehydration therapy, vaccine, phage therapy, probiotics

## Abstract

Cholera infections caused by the gamma-proteobacterium *Vibrio cholerae *have ravaged human populations for centuries, and cholera pandemics have afflicted every corner of the globe. Fortunately, interventions such as oral rehydration therapy, antibiotics/antimicrobials, and vaccines have saved countless people afflicted with cholera, and new interventions such as probiotics and phage therapy are being developed as promising approaches to treat even more cholera infections. Although current therapies are mostly effective and can reduce disease transmission, cholera outbreaks remain deadly, as was seen during recent outbreaks in Haiti, Ethiopia, and Yemen. This is due to significant underlying political and socioeconomic complications, including shortages of vaccines and clean food and water and a lack of health surveillance. In this review, we highlight the strengths and weaknesses of current cholera therapies, discuss emerging technologies, and argue that a multi-pronged, flexible approach is needed to continue to reduce the worldwide burden of cholera.

## Introduction

For centuries, cholera has wreaked havoc on developing countries with poor infrastructure, sanitation, and access to clean drinking water. Cholera also flourishes when normal societal function is disrupted, such as during natural disasters like the 2010 earthquake in Haiti ^1^ or the current refugee crisis in Yemen ^2^. *Vibrio cholerae*, the etiological agent of cholera, is a Gram-negative, rod-shaped pathogen that can cycle between two distinct environments—persistence in brackish-water ponds and infection of the human gut—and it transmits from the environmental reservoirs to the human host via contaminated food or water. *V. cholerae* is highly sensitive to the low pH of the stomach and thus the infectious dose for this bacterium is high at greater than 10 ^8^ organisms ^3^. Those cells that survive the stomach acid eventually colonize the intestinal tract. The toxin co-regulated pilus (TCP) aids in colonization by promoting bacterial microcolony formation. *V. cholerae* then secretes cholera toxin (CT), which disrupts normal ion transport of the gut epithelium, inducing the massive water efflux into the intestine which leads to debilitating diarrhea and vomiting ^4^. Transitioning between biofilm formation and motility during infection is also a key component of *V. cholerae* colonization ^5^.

Out of more than 200 serogroups of *V. cholerae*, only the serogroups O1 and O139 have been the causative agent of current epidemics, and O1 *V. cholerae* is the major infectious agent ^2^. Overall, owing to a lack of TCP or CT, non-O1/non-O139 biotypes do not cause cholera, but there are cases where they do instigate diarrheal symptoms ^6^. The O1 and O139 strains are prevalent in several endemic regions, including Yemen, parts of Africa, Southeast Asia, and Haiti ^2, 7– 12^. Serogroups are subclassified into two major biotypes. The first six pandemics of *V. cholerae* from the years 1817 to 1921 were caused by the classical biotype, whereas the seventh and current pandemic that started in 1961 was caused by the El Tor biotype ^13^. Virtually all modern-day cholera is caused by El Tor, and environmental sampling identifies only El Tor, suggesting that classical biotypes are no longer prevalent. The El Tor biotype can be grouped into the serotypes Ogawa and Inaba, which are the most prevalent serotypes that are causing the pandemics ^2, 14, 15^, and these are used in contemporary vaccines like Shanchol and Euvichol ^16^. Even with modern-day treatments, it is estimated that there are over 3 million cases of cholera with more than 100,000 deaths annually ^16– 18^. The World Health Organization (WHO) public database of annual epidemic cholera cases provides outbreak updates and a summary of worldwide infections ^19^.

**Figure 1.**
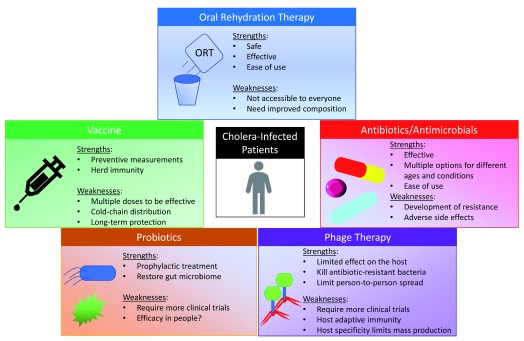
The five strategies to treat cholera. This diagram summarizes the strengths and weaknesses of five different cholera treatments discussed in this review. By considering the strengths and weaknesses of the current therapies and leveraging the diversity of resources and new technologies, a multi-pronged approach could well improve the chances of success in combating cholera infections worldwide and potentially establish cost-effective, pre-emptive solutions more quickly than conventional methods of treatment.

The objective of this review is to describe the current strategies of oral rehydration therapy (ORT), antibiotics, and vaccination which are used to treat and prevent cholera. (See Figure 1 for an overview.) We also highlight novel emerging approaches to treat and prevent cholera—such as probiotic treatment and phage therapy—that have shown success in laboratory conditions but are not yet used in human populations. Because cholera outbreaks often are linked to poor infrastructure, lack of access to clean water, or societal disruptions, our view is that a multi-pronged, flexible strategy is needed to combat these infections, and each of these treatment strategies can meet a specific need to reduce the burden of cholera ( Figure 1).

## Oral rehydration therapy

ORT has a long and interesting history in the field of medicine as a therapeutic to treat acute diarrheal infection. Based on prior knowledge that glucose was essential to facilitate absorption of water from the gut ^7, 20^, the idea of ORT was first attempted in 1964, when US Navy Capt. Robert Phillips used oral glucose saline to successfully treat cholera in two patients in the Philippines ^21^. ORT has since become the most widely used quintessential cholera treatment. Prior to ORT, cholera infections had a mortality rate of more than 50%. However, ORT has treated infection in millions of individuals and saved millions of lives by replacing lost fluids and electrolytes during infection ^7, 20^. This treatment strategy relies on the fact that cholera is a self-limiting infection. Thus, if the patient can survive the massive fluid loss elicited by CT, the infection ultimately will resolve within a few days. ORT has reduced the mortality rate of cholera by more than 97%, and more than 99% of patients on ORT survive *V. cholerae* infections ^14, 22^. Because *V. cholerae* infections cause the intestinal epithelial cells to lose copious amounts of essential electrolytes, conventional ORT prescribed by the WHO contains several vital ions (sodium, chloride, and potassium) and a carbon source (glucose).

Though effective, the constituents of ORT have been studied and modified since its inception. Glucose, one of the components of ORT, can increase the production of CT, the main cause of the severe symptoms associated with the disease ^7^. Kühn *et al*. established that a rice- or starch-based ORT would circumvent this dilemma ^7^. Glucose stimulates Na ^+^ absorption faster than rice starch in the small intestine, so it was possible that a glucose-based ORT would be shorter and more cost-effective. However, even with the slower Na ^+^ absorption rate, the rice-based ORT reduced stool volumes by 36% compared with glucose ORT ^23^. Recently, the rice-based ORT was successfully field-tested to treat cholera in Haiti ^7^. Concomitantly, starch-based ORT is resistant to metabolic degradation in the small intestine, persisting longer than glucose ^20, 23^, and it does not significantly induce CT production ^7^. A starch-based ORT has the additional benefit of stimulating the synthesis of short-chain fatty acids (SCFAs) that can lessen the occurrences of diarrhea by activating the retention of ions by colonic epithelial cells. These SCFAs are produced from starch through fermentation of carbohydrates which are not rapidly absorbed or degraded in the small intestine by the colonic microbiome ^20^. Moreover, since glucose increases ion absorption in the small intestine, these two additives could have a synergistic effect at lessening cholera symptoms ^20^. Though not yet endorsed by the WHO, these alternative carbon-based ORTs exhibit potential benefits to treat cholera and could supplant glucose-based ORT as the major treatment for cholera.

Although modifying the ORT ingredients to achieve optimal Na ^+^ and water absorption in the intestines reduces symptoms, there remain cases where ORT could not keep the cholera symptoms in check. For instance, severe dehydration requires intravenous rehydration. Because ORT is not 100% effective, concurrent treatments such as antibiotics/antimicrobials ^23– 25^ and vaccination ^26^ may be necessary to sustain the reductions in cholera symptoms, as will be discussed in the next two sections.

## Antibiotics/Antimicrobials

The objective of antibiotics to treat cholera infections is to reduce both (1) the time and severity of the illness and (2) the transmission to other individuals. Acute infection with severe dehydration is treated with ORT and antibiotics to produce synergistic efficacy ^10– 12, 16^. Effective antibiotics to treat cholera are doxycycline, azithromycin, and tetracycline. Administration of multiple doses of 12.5 mg tetracycline for 3 days can reduce the duration of symptoms in adults from 4 to 2 days and average stool volume from 21 L to 8 L ^23^. A single dose of doxycycline (300 mg for adults and 6 mg for children) is as effective as multiple doses of tetracycline ^2, 24^. On the other hand, an analysis of significantly more trials with indirect comparisons of tetracycline to doxycycline found that patients who received tetracycline had a shorter duration of diarrhea (by 1 day) while the stool volume reduction was significantly higher ^17^. Likewise, a single dose of 20 mg azithromycin can stop diarrheal symptoms in less than 48 hours—24 hours earlier than with ciprofloxacin ^25^—and decrease vomiting frequency while allowing passage of an average of 36 stools with volumes averaging about 5 L ^2, 24^. Azithromycin is recommended for pregnant women and young children whereas tetracycline is suitable for adults. They are both more advantageous than ciprofloxacin and erythromycin ^1, 11, 12^.

One drawback to antibiotic therapy is that *V. cholerae* O1 and O139 strains have developed resistance to most of the antibiotics that are used. For example, ciprofloxacin, a type of fluoroquinolone that was commonly used in the early 1990s because of its long half-life and high *in vitro* activity, was ineffective in multiple countries with a high burden of cholera infection, such as Haiti and Bangladesh ^24, 27^. This is because O1 and O139 are also resistant to nalidixic acid, which has a mechanism similar to that of ciprofloxacin, and this mechanism confers cross-resistance to ciprofloxacin ^24, 27^. Strains resistant to tetracycline were isolated in several developing countries like Bangladesh, India, Thailand, and Northern Vietnam ^2, 14, 25^, and based on sequencing analysis, the resistance to tetracycline is plasmid-mediated, suggesting that it could continue to rapidly spread in *V. cholerae* populations ^25, 27^. To avoid the development of resistance to these agents, Khan *et al*. recommended taking these medications only when the resistant strains are not prevalent ^27^. Furthermore, *V. cholerae* is evolving new genetic mechanisms to confer resistance to these drugs. Models that predict the emergence of new pandemic strains in heavy-burden, developing countries may be useful for planning future antibiotic treatment strategies, including proper drug allocation, and for elucidating the epidemiology of drug-resistant outbreak strains ^10^.

Although these classes of antibiotics can achieve positive therapeutic effects, it is important to consider the adverse side effects of these treatments. Hypersensitivity reactions are the most common life-threatening side effect of antibiotic treatment of cholera, whereas the irregular cardiac rhythm condition is common only in ciprofloxacin and azithromycin ^24^. ORT and antibiotic therapy function to treat cholera infections but do not prevent patients from acquiring cholera. Therefore, the next intervention that we will discuss that has the potential to limit cholera infections in susceptible populations is vaccines.

## Vaccines

The WHO advocates the use of oral cholera vaccines (OCVs), including both live-attenuated and inactivated oral whole cell (WC) vaccines, in endemic areas or during outbreaks as a transient protection since they have been shown to be effective in combination with other correlative treatments, including antibiotics, ORT, and health management ^17, 28^. OCVs principally stimulate mucosal immunity mediated by antibodies, particularly IgA, against the pathogen. These antibodies are directed against antigens such as O1-specific polysaccharide and CT ^28^. Although IgA has limited systemic circulation (~6 months), the memory B cells that are responsible for preventing cholera infection persist and can quickly expand and differentiate into plasmablasts and eventually the plasma cells, which can reseed protective antibodies upon antigen-contact activation ^28^. Moreover, OCVs could provide herd immunity to unvaccinated adults, but the effect in unvaccinated children requires further study ^29^.

One widely used WC strain vaccine is Dukoral (CTB-WC), which contains inactivated/dead *V. cholerae* O1 (El Tor and classical biotypes) with the addition of recombinant B subunits of CT (CTB) ^12, 16, 30, 31^. The effectiveness of Dukoral is between 55% and 88% ^17^, and it is intended for travelers but—owing to its short period of usability, high cost, and its requirements for cold-chain circulation—not for populations in endemic regions ^9, 25^. Dukoral can provide protection from infection for 2 years in vaccinated individuals above the age of 5, but it is effective for only 6 months between the ages of 2 and 5 and requires at least two doses to be effective ^16^. Unlike Dukoral, Shanchol and Euvichol are WC vaccines composed of inactivated O1 Inaba, O1 Ogawa, and O139 strains, but these vaccines do not contain CTB ^16, 32^. The efficacy of Shanchol is about 65% protection based on a 5-year study ^17^. In clinical studies in the Philippines, Euvichol was effective in adults and children ^18, 32^. Shanchol and Euvichol are intended for all patients who are at least 1 year old but not for pregnant women ^16, 18^. Furthermore, there are two variations of Euvichol (with or without the preservative thimerosal), both of which show no significant difference in protection ^32^. These WHO prequalified inactivated vaccines can provide protection against cholera for at least 3 years and are not available in the US ^4^.

Aside from the inactivated *V. cholerae* vaccines, the oral live-attenuated vaccine Vaxchora (CVD 103-HgR) is a US Food and Drug Administration–approved, single-dose vaccine that protects against either the Inaba or Ogawa serotype and contains CTB from both classical and El Tor biotype ^2, 30, 33^. Owing to the robust, rapid cell-mediated protection of Vaxchora, its efficacy against cholera is estimated to be around 90% post-vaccination and 80% 3 months post-vaccination in travelers to high-risk cholera areas ^2, 30, 33^. The next steps for Vaxchora development are to evaluate its safety and effectiveness in cholera-endemic populations and to optimize the preparation of the vaccine since it relies on cold-chain shipping and water mixing, which are problematic for distribution in some endemic or disrupted regions ^33^.

Several alternative forms of vaccines are being developed and these include a combined outer membrane vesicle (OMV) vaccine against *V. cholerae* and *Escherichia coli* that has been shown to induce a strong immunogenic response ^12^, a genetically manipulated form of live *V. cholerae* without the diarrheagenic factors to mediate probiotic-like protection from cholera ^34^, and antimicrobial glycoconjugates, particularly the lipopolysaccharide epitopes across different serotypes (Ogawa and Inaba) ^35^. These alternative forms of vaccines are not yet in clinical trials.

Effectively using vaccines to prevent or curtail cholera outbreaks relies heavily on epidemiological research as different endemic regions need distinguishing vaccines to target the divergent circulating strains. Moreover, ideal cholera vaccines will not be dependent on cold-chain shipping. In addition to these three treatments, which are currently used, new approaches to prevent or treat cholera infections are emerging.

## Probiotics

An emerging concept in microbiology is the ability of the host microbiome to prevent or limit infections. A relatively new concept for *V. cholerae*, this idea is beginning to be explored as a treatment or prevention for cholera infections. Owing to the excessive fluid accumulation, *V. cholerae* elicits severe disruption to the gut microbiome during infection such that the majority of bacteria found in the characteristic rice-water stools are *V. cholerae* ^36^. Furthermore, the type VI secretion system of *V. cholerae* can deliver effector toxins to the gut microbiome or modulate host cells themselves, both of which alter the gut microbiota to facilitate colonization ^37, 38^. For these reasons, restoration of the gut microbiome or prevention of colonization through probiotic treatment is a promising new approach to treat cholera infections.

Several bacterial species have been shown to dispel or suppress cholera infections. *Ruminococcus obeum* increases in its relative abundance after *V. cholerae* infection of mice and restricts *V. cholerae* colonization by disrupting its quorum-sensing system ^39^. Interestingly, *R. obeum* is one of the species in the human gut microbiome whose abundance positively correlates with recovery from cholera ^39^. Co-culture of *V. cholerae* with *Lactobacillus rhamnosus* GG or *Bifidobacterium longum* 46 decreases CT production *in vitro* ^40^. One study engineered a probiotic strain of *Lactococcus lactis* that increases the production of lactic acid upon detecting the quorum-sensing signals of *V. cholerae*, thus decreasing the pH of the surrounding medium to reduce *V. cholerae* during co-culture ^41^. Another experiment engineered an *E. coli* strain to mimic the CT binding ganglioside on its surface and this strain reduced the symptoms of a *V. cholerae* infection by decreasing the free CT ^42^. The culture supernatant of a fecal *Lactobacilli* strain was exploited to disturb a *V. cholerae* biofilm by increasing the pH to potentially reduce stress in the gut ^43^. Lastly, *E. coli* was demonstrated to decrease the colonization of *V. cholerae* when co-cultured with glucose in a zebrafish infection model by lowering the pH ^44^. This finding is intriguing as it suggests a possible synergistic effect between probiotics and glucose-based ORT ^44^.

Probiotics often are taken with antibiotics and various other drugs, including anti-inflammatory adjuvants, but the adverse side effect of the drugs on the probiotics warrants further testing ^43^. Nevertheless, probiotics that reduce cholera may limit antibiotic-resistant *V. cholerae* strains by reducing the quantity of antibiotics used ^41, 42^. Moreover, probiotics could serve as a better treatment in regions where cold-chain vaccine preparation is not feasible and clean water supply is not available ^40^.

## Phage therapy

Another novel treatment for cholera involves the therapeutic use of lytic bacteriophages. Phage therapy has been used for decades in Eastern Europe and Russia and, with the emergence of antibiotic-resistant bacteria, has been developed and used to treat infections caused by *Pseudomonas*, *Salmonella*, and *Staphylococcus* ^45^. Phage therapy has many advantages over antibiotics. For example, phages are able to kill antibiotic-resistant bacteria, the amount of phages increases proportionally to the number of infecting bacteria, and the phages exert a minimal impact on the resident microbiome ^45^. This treatment strategy is inspired by the natural life cycle of *V. cholerae* in which blooms of the bacteria during outbreaks are followed by the expansion of lytic bacteriophages, primarily ICP1, ICP2, and ICP3, which ultimately reduce the population of viable bacteria ^46, 47^.

A study in an infant mouse model has shown that a cocktail of the three ICP virulent bacteriophages could effectively reduce the *V. cholerae* load after challenge in a dose-dependent manner because of the phage’s fast replication and ability to kill the bacteria ^48^. Although it shows promising results in animal models, phage therapy for cholera requires more optimization for its effectiveness and timing to be advanced to clinical trials ^48^. Phage therapy could also be used to limit person-to-person spread as even small numbers of these lytic phages could rapidly expand during *V. cholerae* infections.

Although phage therapy has many promising characteristics, it also has potential drawbacks. During phage therapy, the host’s adaptive immune system can generate phage-neutralizing antibodies that could inhibit their ability to lyse the targeted bacteria *in vivo* or prevent subsequent treatments ^45^. As with antibiotics, *V. cholerae* will evolve resistance to phage infection and thus the most likely application of phages would require a lytic phage cocktail that would necessitate multiple independent mutations for resistance. Because of the intricate connection between *V. cholerae* and lytic phages, this bacterium encodes molecular defense mechanisms to limit phage infection ^49^. Owing to host specificity, which has a significant impact on treatment development and testing, mass production and distribution of phage therapy, are not yet practical ^50^. Before phage therapy is validated as a cholera therapeutic, there must be an assessment of the immunological response to phages and efficacy during cholera outbreaks.

## Conclusions

The biggest challenges to treat *V. cholerae* are the inherent complications in endemic or disrupted regions, including economics, natural disasters, wars, national security, and poor infrastructure. Because of these challenges, a multi-pronged approach that is flexible to the specific demands of a current outbreak is needed to treat cholera. ORT has been and will continue to be a front-line defense to save patients already infected with *V. cholerae* because it is cost-effective and easy to use and combining antibiotics with ORT clearly reduces the severity of disease. Additionally, the WHO has prequalified Dukoral, Shanchol, and Euvichol as current cholera vaccines, although there is a worldwide deficiency of these vaccines ^2^. Increasing the availability of these vaccines could have a significant impact in reducing infections during an outbreak. Whether probiotics or phage therapy can work synergistically with ORT, antibiotics, or vaccines to treat or—preferably—to prevent cholera infections remains to be tested in the field. Another approach to control cholera is the development of anti-virulence compounds that inhibit the expression of virulence factors, thereby protecting the host from colonization by *V. cholerae.* Such compounds include virstatin ^51^, a conjugated form of linoleic acid ^52^, and synthetic compounds that resemble folded fatty acids ^53^. Further development of these anti-virulence therapeutics requires testing these compounds during human infections and assessing their practicality to treat cholera outbreaks. Ultimately, health surveillance plays a critical role in preventing outbreaks by directing proactive countermeasures during emergencies. A global commitment to reduce pandemic cholera requires devising better methods to quickly identify outbreak strains, recognizing the best treatment option for the given strain, and developing new therapies that are not dependent on cold-chain systems or clean water. The combination of current treatments with new therapies has significant potential to further combat the centuries-old human scourge of cholera.
